# A Low Power Consumption Algorithm for Efficient Energy Consumption in ZigBee Motes

**DOI:** 10.3390/s17102179

**Published:** 2017-09-22

**Authors:** Daniel Vaquerizo-Hdez, Pablo Muñoz, María D. R-Moreno, David F. Barrero

**Affiliations:** Intelligent Systems Group, Computer Engineering Department, Universidad de Alcalá, 28805 Alcalá de Henares, Spain; pablo.munoz@uah.es (P.M.); malola.rmoreno@uah.es (M.D.R.-M.); david@aut.uah.es (D.F.B.)

**Keywords:** WSN, duty-cycling, ZigBee, battery maximizing, data collection

## Abstract

Wireless Sensor Networks (WSNs) are becoming increasingly popular since they can gather information from different locations without wires. This advantage is exploited in applications such as robotic systems, telecare, domotic or smart cities, among others. To gain independence from the electricity grid, WSNs devices are equipped with batteries, therefore their operational time is determined by the time that the batteries can power on the device. As a consequence, engineers must consider low energy consumption as a critical objective to design WSNs. Several approaches can be taken to make efficient use of energy in WSNs, for instance low-duty-cycling sensor networks (LDC-WSN). Based on the LDC-WSNs, we present LOKA, a LOw power Konsumption Algorithm to minimize WSNs energy consumption using different power modes in a sensor mote. The contribution of the work is a novel algorithm called LOKA that implements two duty-cycling mechanisms using the end-device of the ZigBee protocol (of the Application Support Sublayer) and an external microcontroller (Cortex M0+) in order to minimize the energy consumption of a delay tolerant networking. Experiments show that using LOKA, the energy required by the sensor device is reduced to half with respect to the same sensor device without using LOKA.

## 1. Introduction

The Wireless Sensor Networks (WSNs) are systems to exchange information between different places without wires [1]. Innovations in microelectronics have enabled the development of low-cost and low-power multifunctional sensors that are small in size and communicate untethered in short and medium distances, leading to a large number of applications.

Generally speaking, a mote is composed of a microcontroller, a radio and one or more sensors (eventually, actuators). Sensors measure their environment and the measures are transmitted by a radio. All these operations are controlled by a microcontroller, which implements the control logic and some basic data processing.

The goal of a typical WSN is to cover a location with tiny motes leveraging the collaborative effort to improve the perception of the studied place. Each device usually measures variables about the environment where they are deployed and they share these variables between them integrating a powerful tool to collect information.

The WSNs improve traditional sensors for some applications since the communication protocols are carefully designed for quick, reliable and energy-efficient communication. The motes transmit sensed information to other motes when needed. Then, the data are processed and fused in the collector mote. Also, the position of the motes are not pre-determined; the communication protocols enable random deployment of sensor motes selecting the shortest way to transfer data to the collector mote due to the ability of motes to relay information. In other words, the WSNs have self-organizing capabilities, making their deployment much easier.

Nowadays, this technology is used in many applications like telecare systems [2], automotive [3], domotic [4], industrial processes [5], smart cities [6], military [7], and others. For instance, the state of a dependent person can be monitored by a WSN, recognizing and preventing crisis in the home, assisting daily routines and providing awareness of daily life and long-term trends [8]. Other related projects use WSN technology to measure health variables into different places such as nursing homes, hospitals or homes [9]. Other WSN can also be used to detect filled garbage containers along an area of a city to optimize the route of the garbage trucks [10]. Another example is fire detection in forests; deploying motes with fire sensors in a WSN along a forest enables to detect small blazes before a big fire [11]. In substance, the WSNs can provide a better understanding of the environment offering a good solution to gather information that will be assessed by an intelligent system.

The energy consumption of each mote in a WSN highly influence the autonomy of the WSNs applications. Usually, the motes are powered by batteries and thus can be placed anywhere independently in the electricity grid. It means that the operation time is fixed by the time that the battery can power the mote. Maximizing the operation time is critical in many applications where the costs of recharging the batteries are important. For instance, in the case of a telecare system, the dependent people often forget to recharge the motes and, therefore, the WSN will not work properly. In the same way, in the detection of forest fires, the WSN covers large area where often there is not an electrical grid, making the batteries recharge a very expensive task. In summary, energy saving is a critical aspect in WSN design.

In this context, a growing demand of WSN systems requires sophisticated skills to handle the energy consumption to maximize the operation time of the WSN. The efficient usage of the communication protocol, transmission data interval and power consumption modes of the motes electronics components have great impact. We can distinguish two main components in each mote affecting the power consumption: the radio and the microcontroller. Depending on the emission power of the antenna and the communication protocol, the mote will consume more or less energy, affecting the battery life.

The researches struggle to find strategies in order to maximize the battery time. These strategies are focused on the hardware, the route protocols and the data treatment. Also, all these strategies are combined with the newest WSN approach in order to join efforts. First, choosing a compatible hardware. Second, selecting the route protocol depending on the path of the information. Last, minimizing the data size to send.

Once the strategy is chosen, there are three actions of each mote that influence the energy consumption: sensing, processing and transmission. Each action has its own consumption; the sensing energy consumption depends on the sensors of the mote and the associated hardware. The processing energy consumption depends on the microcontroller and the data treatment strategy. The transmission energy consumption depends on the antenna, the radio transceiver and the path.

The sustainable operation of battery powered wireless embedded systems is a key challenge. Mote energy harvesting, particularly in the energy consumption of each mote, has emerged as a viable technique to improve the time between battery charges. The management of the power modes directly affects the energy usage and the storage decisions. The ability of the system to modulate its power consumption by selectively deactivating its sub-components also impacts the overall power management architecture. Taking this into account, harvesting technologies hardware depends on the batteries of each sensor motes. Particularly, the cost of the hardware depends on the batteries size. This paper is useful in reducing the power consumption and therefore, the batteries size and the cost of the harvesting hardware.

Considering that the energy consumption optimization minimizes the energy harvesting, we proposed an algorithm that exploits the selected hardware and the communication protocol in order to achieve the minimum energy consumption at a given time and therefore, minimize the energy harvesting efforts.

Taking that into account, the contribution of this paper is a LOw power Konsumption Algorithm (LOKA) that controls the program flow of a mote and reduces its energy consumption. It controls the energy mode of each electronic device of the sensor mote based on the state of the communication  protocol. LOKA forces a low power mode in the idle devices of each mote when they are not working, and changes it when they need more computational power. Then, the reduction of energy consumed will be exponential depending upon the transmission data interval and on the operation time of the WSN application.

LOKA is the result of the problems encountered during the testing of a WSN deployed in homes of dependent people for tele-assistance . During the tests, we needed to monitor the people movements and different phenomena of their daily life in conjunction with an assistant telecare robot. We realized that the battery life was very short and we could not count on the monitored people (since they had Alzheimer’s disease) for charging the motes. In this sense, with LOKA, we were able to monitor the dependents for more than two months with a single charge instead of a few days as happened initially.

This article is organized as follows. Next section introduces the state of the art of solutions in reducing the motes energy consumption. After, Section 3 provides: (i) the reference framework where LOKA can be implemented; (ii) the communication protocol used and (iii) the hardware considerations for LOKA. Then, LOKA is described in detail in Section 4. Next, Section 5 presents the experiments divided in two blocks: the first block shows the design for the mote where LOKA was embedded for the experiments, while the second block explains the method to measure the energy and provides the values obtained in our experiments. Finally, some conclusions are outlined.

## 2. State of the Art

A fundamental problem of WSN systems lies in the relatively high energy consumption. The most demanding component in energy terms is perhaps the radio, usually the batteries are not big enough to support long time and long distance communications because of the needed emission power. The  microcontroller also affects the consumption, specially when it is executing a computation intensive task. Notwithstanding, energy consumption for communications is much higher than for computing.

In order to reduce the energy consumption, some researches are focused on the data transmission  [12,13]. Rohini et al. compared different communication protocols with their own protocol based on mote positions and subdivision into regions to improve the data transmission energy consumption. Yan et al. used a node cut strategy to dynamically generate filtering tuples that have little computational overhead when collecting query results, instead of issuing queries with filters. This reduces the comparison frequency and the computation time.

Other researchers optimize the energy efficiency by using optimal cluster head selection, forming chains of nodes or balancing the load, i.e., focusing on the topology of the WSN. Shall et al. [14] suggested implementing an optimized solution to the problem of arranging objects in an scalable IoT. Sudhanshu et al. [15] discussed about the use of different protocols for clustering in WSN providing a complete vision of the problem.

Likewise, there are researches targeting in minimizing the energy consumption taking into account the time between transmissions. Bruno et al. [16] suggest an energy-aware adaptive sampling method that allows each mote in the network to adapt the sampling rate to its sensors according to the available energy in the system.

Moreover, there are power management schemes in the motes that attempt to control the radio depending on the state of the data transmission, called wake-up protocols. These protocols are grouped into three categories: duty-cycling, non-cycled wake-up and path reservation wake-up protocols [17] that can be available in the Media Access Control (MAC) layer.

The duty-cycling protocols are mechanisms that turn the radio on and off to conserve energy [18], meanwhile a sleep-wake up cycle is employed to save energy reducing the energy consumption in idle listening, it increments the waste power due to control packet overhead and collisions. Non-cycled wake-up protocols use a low power wake-up radio that is active all the time or use a passive circuit that should be powered from the wake-up signal, which is only able to receive the wake-up message [19]. Finally, path reservation wake-up protocols exploit parallel data communication and channel reservation [20]. In this case, communications are in multi-loop links and the wake-up signal is independent to data transmissions using separate radio channels.

In duty-cycling mechanisms, depending on the tolerance of the scenario, i.e., message delay, collision rates and control packet overhead (synchronization or asynchronous transmission), duty-cycling may work efficiently or not. As a consequence of the different scenarios, there are different approaches of duty-cycling: synchronous, asynchronous or semi-synchronous approach.

In the synchronous approach motes exchange synchronization information through the network to archive a degree of synchronization [21,22]. This approach is useful when each mote has its own data exchange time window. The semi-synchronous approach groups neighbor motes in synchronous clusters that exchange information asynchronously among them [23,24]. Finally, in the asynchronous approach, instead of exchanging synchronization information, multiple techniques are implemented to avoid the packet overhead and collisions such as preamble sampling where all motes go to sleep asynchronously and often wakes up for checking channel activity [25]; the receiver-initiated approach where the sender waits for a beacon from the receiver [26]; or the random duty-cycling method, where motes are in sleep mode and wakeup randomly [27]. These duty-cycling mechanisms are integrated in operating systems such as *TinyOs* [28] and *Contiki* [29] yet these operating systems do not work in platforms where there is an external microcontroller like in our LOKA approach.

Other techniques can be grouped in Low Power Listening (LPL) and Low Power Probe (LPP). In LPL if the sender energy level is above a predefined threshold, the receiver stays awake to receive the potential packet then, the receiver sends an ACK. In LPP, each receiver periodically wakes up to send a probe, the sender meets the receiver by successfully decoding the probe and send it the information. LPL and LPP suffer performance degradation in noisy environments with signal interference. In this way, Xialong et al. [30] implement *ZiSense*, a technique that minimizes the false positives due to interferences measuring the Received Signal Strength Indication (RSSI). This approach can be included into LOKA yet it is not the aim of our paper since LOKA focuses on delay buffer transmissions.

Furthermore, duty-cycling approaches can be divided depending on the mote type that implements it. Some papers focus on the coordinator such as the work of Giovanni [31] that incorporates a fuzzy logic control in the coordinator device (receiver) to predict when an issuer sends a message. This approach allows maximizing the coordinator sleep time when it is idle. A priori, this technique will not fit in our approach since it needs a lot of information or many receiver packets to allow the use of fuzzy logic and LOKA works in motes that receive few packets since these motes are sensor motes.

Therefore, the goal of this research is to contribute to minimizing the power consumption by means of a duty-cycling algorithm, LOKA, in a sensor mote composed by a Cortex M0+ microcontroller and a ZigBee module included in a tiny embedded prototype. For that purpose the algorithm takes into account the transmission time interval and the power modes of the devices attached to the mote.

## 3. LOKA Scenario

This section presents the scenario where LOKA was deployed. It is composed of the reference framework, the communication protocol and the hardware requirements where LOKA will be implemented in order to decrease the energy consumption of the WSN. First of all, the reference framework where LOKA was developed is described. Then, we discuss the chosen communication protocol decisions in order to handle the operations of each device that form the mote. Finally, the  hardware connections are showed in order to support LOKA.

### 3.1. Reference Framework

The tele-assistance domain where LOKA was developed presents the next characteristics identified as interesting and shared in common for multiple WSN applications:

- High safety. The information gathered by the WSN includes sensitive data so, the WSN must be protected against misappropriation.
- Robustness. The WSN should be recovered if there are some interferences.
- Independence from the power grid. They must integrate a battery because the motes should be located anywhere.
- Low power consumption. To improve the autonomy in terms of energy consumption, each mote has to be as efficient as possible.
- Adequate coverage. The WSN should cover different areas in order to be spread out in different  applications.

For WSNs, wireless hosts do not need a fixed infrastructure. The motes can communicate among themselves without central control and the communication between two hosts in an WSN can be done in a multi-hop manner as Josh et al. [32] pointed out for different scenarios, creating mesh networks. With this in mind, the developed algorithm reduces the energy consumption in a sensor mote, independently of the network topology.

Apart from those features, as it is common in the Zigbee Application Support Sublayer (APS), three types of ZigBee Device Object (ZDO) coexist in the network. The first one is the coordinator that forms the network and controls it. The second one is the end-device that senses the values of the phenomena. The third one is the router that allows raising the coverage of the network. Each mote has only one ZDO and therefore the term mote is equivalent to ZDO.

It is worth saying that end-devices cannot route packets [33]. They can read packets and send them but they cannot receive packets from a sender to a receiver as a relay node. This is a peculiarity of the ZigBee protocol and it cannot be changed without redesigning the protocol. In this sense, the end-devices only read the packets that have the same device address number. This is not a problem since the ZigBee WSNs can be multi-hop placing router devices along with end-devices.

LOKA uses the end-devices inasmuch as they have the characteristic to implement a duty-cycling mechanism allowed by the Zigbee protocol. The end-devices go to sleep if they are not needed and LOKA controls their duty-cycling mechanism in conjunction with another duty-cycling mechanism implemented in the external microcontroller.

Thus, our proposal is to include a coordination ZDO into the collector mote and to include an end-device ZDO into each sensor mote. The difference between a coordinator ZDO and a collector mote is that the coordinator mote is the one that controls the network, meanwhile the collector is the final receiver of the data produced by the end-device motes. More in detail, when the coordinator ZDO starts up, it scans all channels, starts a network on a channel with low or without activity and controls the joining and leaving of other motes in the channel. Therefore, the coordinator ZDO should always be powered on. By its side, a collector mote is the one that receives all information produced, being the final receiver of all data transmissions. We have decided to incorporate a single collector since the collector was a gateway for an Internet database.

The difference between a sensor mote and an end-device is that the first one implements the functionalities and the hardware to sense values of phenomena, meanwhile an end-device exploits the capabilities of modern WSN protocols, such as ZigBee, to reduce the energy consumption implementing a duty-cycling mechanism. The end-device is usually combined with a sensor mote that performs periodical operations or triggered by events.

Our problem goes along with a data collection scenario, where the sensor motes monitor variables at a given rate or triggered by an event, and report the data to a collector mote. Thus, the collector mote will receive all the information if a certain event happens or just periodically. LOKA is deployed in each sensor mote and works independently of the receiver or the sender (coordinator, router or end-device), but in our dependent-persons application the final receiver was the coordinator. It is worth mentioning that LOKA supports the receipt of packets in the end-device motes.

Often, an external microcontroller is included into the motes with the aim of performing small data processing since the commercial ZigBee devices only incorporate the necessary resources to implement the protocol. In our approach, we have considered this option, and therefore, we minimize the consumption of both devices (the external microcontroller and the ZigBee device) with the aim of reducing the WSN energy consumption as a whole. This energy consumption minimization is performed by LOKA.

**Figure 1:**
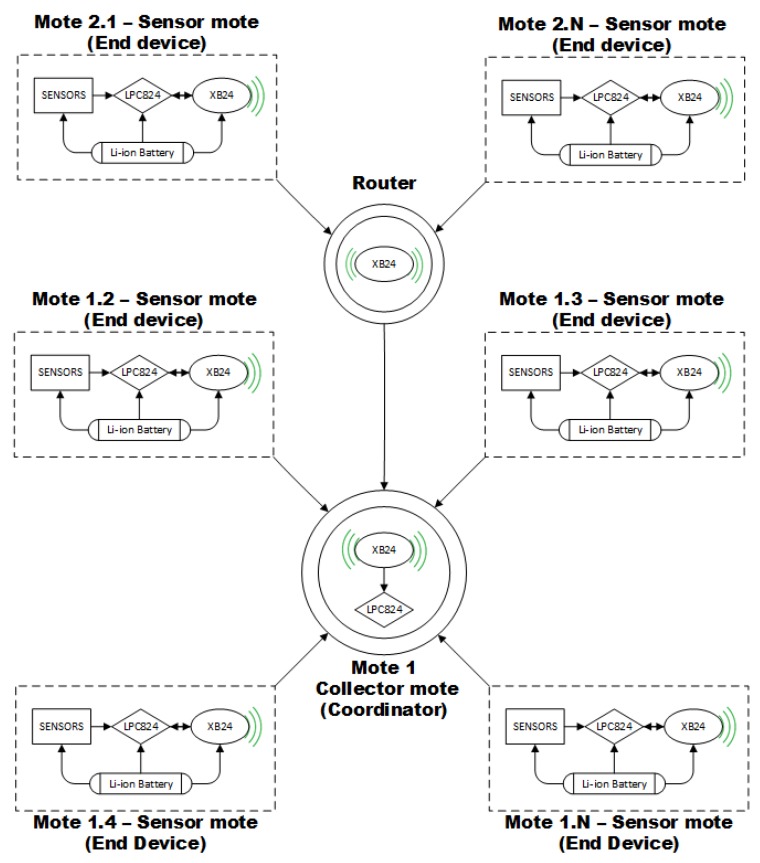
Reference Framework. It is composed of the collector mote, the router motes and the sensor motes. The sensor motes send the data information to the collector and then the collector processes it.

Figure 1 illustrates our reference framework composed of N sensor motes sending information to a collector mote that analyses it. The sensor motes are composed of a Cortex M0+ microcontroller (LPC824), a ZigBee module (XB24), a radio, a battery and sensors to obtain the phenomena information. Thus, the microcontroller commands the sensor mote to take samples and then, it activates the radio to send these samples to the collector mote using a communication protocol. The collector mote does not have sensors because it is in charge of receiving and analyzing the information; it only integrates a radio, a microcontroller and a battery. Thus, the microcontroller receives and processes the samples taken by the sensor motes while maintaining the right operation of the sensor network.

While it is true that the ability to route packages in duty-cycling state of the art is an issue, the Zigbee protocol precludes to use end-devices as relay nodes. The network coverage area is often much larger than the radio range of single end-device mote(s). So in order to reach some destination node we can use route device motes as relays in a multi-hop communication.

However, we have not focused on how to route packages in the duty-cycling mechanism. Instead, we want to control the end-device in conjunction with the power modes of an external microcontroller implementing two duty-cycling mechanisms in parallel, sleeping and waking up the end-device and the external microcontroller with the aim of reducing the energy consumption of a sensor mote.

### 3.2. Communication Protocol

In terms of wireless operation, over the last few decades standards for low-power wireless communications have been developed to cope with the requirements of different applications. One of them is the IEEE 802.15.4 standard published in 2003 for Wireless Personal Area Networks (WPAN) or the ISO/IEC 1800-7:2009 standard, both for the physical and data-link layer, MAC. Both standards define the lowest layer, meanwhile protocols like ZigBee, RPL (IPv6 routing protocol for low-power and lossy networks), Constrained Application Protocol (CoAP), Time-Slotted Channel Hopping (TSCH) and DASH7 alliance mode (D7AM) specify the application layer.

In that way, the IEEE 802.15.4 standard is our choice because it is more suitable for periodic data gathering scenarios as suggest by Xavier Vilajosana et al. [34]. Also, in conjunction with the IEEE 802.15.4 standard we have chosen the ZigBee protocol for the application layer. We could have used TSCH instead of ZigBee, but at the use case presented in this paper the TSCH commercial devices were not yet available on the marketplace into commercial devices.

Thus, ZigBee is the lowest power consumption protocol and the best in terms of reliability commercial devices. ZigBee is a wireless communication standard managed by the ZigBee Alliance based on the IEEE 802.15.14 standard, providing a very low consumption if idle mode. Also, it has flexible network topology, allowing multiple configurations. The scalability is achieved by static and dynamic star and mesh topologies. Our proposal is to include a ZigBee device into each mote type.

The ZigBee protocol fulfills the high safety requirement since the Advanced Encryption Standard (AES) can be used for the communication. The information packets sent by the sensor motes are encrypted by the emitter and they are only decrypted into the final receiver with a password. Moreover, the ZigBee network is determined by a specific ID that all devices have to know if they want to join it.

The ZigBee’s characteristics are not modified by the algorithm since LOKA only uses the ZigBee protocol without any internal change ensuring that the coordinator configurations allow the end-device configurations. The packet loss rate, the node connectivity errors and the transmission throughput of the ZigBee protocol depends on the protocol, the radio and the environment [35,36,37] and LOKA does not modify them.

### 3.3. Hardware Specifications

LOKA is in charge of commuting between the different power modes of each device of a sensor mote through the microcontroller to implement a duty-cycling mechanism. Thus, we need to know the connection topology of a sensor mote in order to know the energy consumption magnitude. In this regard, this section explains all electronic components that LOKA takes into consideration.

**Figure 2:**
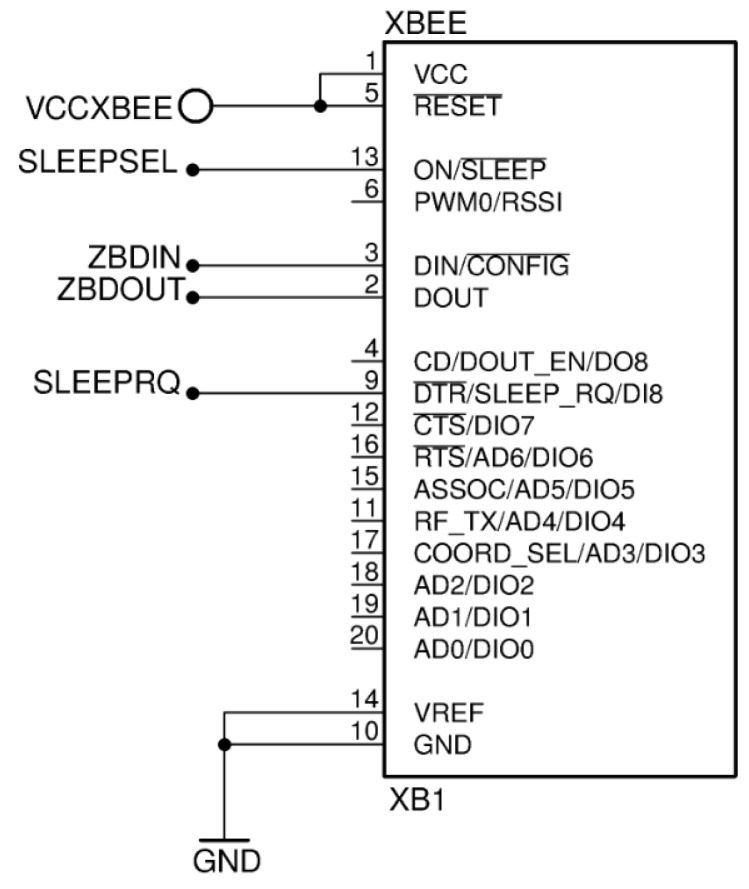
XB24-Z7WIT-004 socket.

Therefore, our choice to the radio module is the ZigBee module XB24-Z7WIT-004 (XB24) of Digi International whose diagram is in the Figure 2. XB24 allows distributed nodes in subnets of motes using an antenna of 2 mW and a sensitivity of 40 m in indoor environment and 120 m in outdoor environment. These modules consist of a memory and a microcontroller that implement the communication protocol, one GPIO to establish the communication between the module and the outside and a radio suited to provide emissions in 2.4 GHz non-licensed band of the industrial, scientific and medical (ISM) radio band standard.

With regards to the XB24 connection, Figure 2 shows the basic socket diagram where it will be deployed. XB24 can receive the sleep or wake up order to change its power mode through SLEEPRQ pin, and XB24 indicates its mode consumption (sleep or active) through SLEEPSEL pin. ZBDIN and ZBDOUT are the TxD/RxD UART protocol pins that implement the communication between an external microcontroller and the XB24.

**Figure 3:**
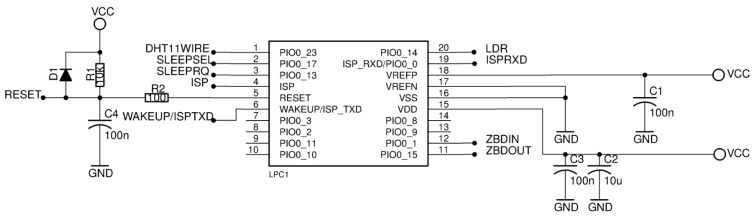
LPC824 microcontroller connection circuit into the sensor mote.

Outside the XB24, the Figure 3 shows the associated external microcontroller LPC824. This  microcontroller controls the power mode of the XB24 module, takes samples from the associated sensors if necessary and execute the control of the information flow between the sensors and the XB24 achieving an effective use of the energy consumption by means of the LOKA.

LPC824 is an 32-bit ARM Cortex M0+ core microcontroller of NXP Semiconductors. We have chosen it because, nowadays, Cortex M0+ is the most energy-efficient processor available: 9.4 µW/MHz. Also, it implements various energy saving modes consumption that LOKA exploits to reduce the sensor mote power consumption.

With regard to the support circuit of the sensor motes, Figure 3 shows the design of the LPC824 microcontroller circuit for our study case. It is composed of the LPC824 surface mount pad, capacitors C1, C2 and C3 recommended by the manufacturer and a reset protection circuit formed by diode D1, capacitor C4, resistor R1 and resistor R2. External clock has not been included in the circuit, instead we use the internal 12 MHz clock. The microcontroller has been connected to the XB24 by a UART and two digital pins to control it: SLEEPSEL and SLEEPRQ. Also the LPC824 can be connected to some sensors to take samples, for instance, by the pins number 1, 6 and 20. Finally, there is an In System Programing (ISP) wire bus connection to upload the control program. This configuration ensures that the LPC824 can do the three required operations: read sensors (by sensor pin connections), control the operation power mode of the XB24 (by SLEEPSEL and SLEEPRQ pins) and send data to the XB24 (using the UART).

Finally, the hardware specifications for the collector mote are the same to the sensor mote except that the collector mote has no sensors attached nor digital pins to control the XB24, because its XB24 will always be in active mode since the collector mote matches with the network coordinator and it must control and manage the security of the WSN.

## 4. LOKA: LOw Power Konsumption Algorithm

This section introduces LOKA, an algorithm to optimize the energy consumption in the above WSN scenario. LOKA is based on the efficient use of power modes of the XB24 and the LPC824. Knowing the transmission data interval of a sensor mote, LOKA selects the adequate power mode in order to save energy when the mote does not require high computing power. LOKA is implemented in the sensor motes since an end-device element can work in different consumption modes: idle, taking samples or transmitting information. However, the coordinator is always in active mode because it must be available to receive data from any end-device while keeping up the network. Thus, LOKA is not implemented in the collector mote.

Two devices of the sensor mote are taken into account in LOKA: the LPC824 microcontroller and the XB24 module. Both come together to optimize the system consumption and they need to agree in the flow of the program control. In order to optimize the energy consumption, both devices should be sleeping when there is no need of processing or sending data. Thus, these devices are in an idle state until a periodical timer wakes them up or when an alarm event is detected.

While it is true that duty-cycling mechanisms are integrated in ZigBee devices [38], many ZigBee commercial modules need an external microcontroller to manage them. In this sense, to the best of our knowledge there is no algorithm o mechanism that allows deploying a duty-cycling mechanism into two devices (ZigBee module and external microcontroller) at the same time.

The novelty of LOKA is the management of a two duty-cycling mechanism in only one algorithm using a commercial end-device module of the ZigBee protocol and an external microcontroller. The proposed algorithm controls the sleep time of both, the end-device and the microcontroller depending on the network needs. Furthermore, LOKA handles the delay buffers of the ZigBee module, enhancing the energy consumption. This improvement is a new approach in newest delay tolerant networking protocols.

Regarding to the LPC824, there are four power consumption modes that can be activated by the System Control Register (SCR): *Sleep*, *Deep-sleep*, *Power-down* and *Deep power-down*. These modes power off different mechanisms of the LPC824 in order to consume less energy. The Sleep mode only affects to the CPU. The *Deep-sleep* and *Power-down* modes affect to the CPU and all the selected peripherals with the following difference: in *Deep-sleep* mode the flash memory is in standby mode, while in *Power-down* mode it is powered down. Finally, *Deep power-down* mode affects all system except for the general purpose registers in the power management unit and the self-wake-up timer. In this mode, the LPC824 reboots when it wakes-up.

In this way, the *Deep-power* down mode is the most efficient low power consumption configuration supported for our scenario because the sensor mote needs to power down all possible devices without rebooting to minimize the energy consumption. All peripherals and memories will be configured to power down in *Power-down* mode, except the following ones:

- Brown-out detection (BOD). It is used to send an interruption when a low battery level is detected.
- Wake-up time (WKT). It is used to generate a cyclic interruption in order to take samples and send them to the collector mote periodically.
- Wake-up pin IRQ. It is used to send a interruption when an alarm event is detected.
- Brown-out IRQ. It corresponds to the active interrupt handler when the brown-out is detected.
- WKT IRQ. It corresponds to the active interruption handler when the wake-up time ends or a signal is detected in wake-up pin.
- WKT low-power 10 kHz clock source. A low power clock is active to synchronize the above elements in *Power-down* mode.

Once the LPC824 is in the *Power-down* mode, it wakes up when one of the following conditions is satisfied:

- The voltage supplied is less than 3 V. Then, an IRQ occurs on BOD.
- End of the WKT count. Then, an IRQ occurs on WKT.
- A falling edge in the WKT pin. Then, an IRQ occurs by the associate digital input-output (DIO)  pin.

Related to the XB24, it implements two types of power modes to reduce the consumption: *Pin  sleep* and *Cyclic sleep*. These sleep modes can be configured through a voltage level in the first mode or through AT commands in the second mode. In this way, our choice is *Pin sleep* mode because the LPC824 could command to the XB24 the time to change from sleep mode to active mode or vice versa. Thus, the Sleep Mode Register (SMR) of the XB24 is set to Pin Hibernate option to support it and LPC824 is connected to the Sleep_RQ pin of the XB24 to control its idle or activation mode. At the same time, LPC824 is connected to the On/Sleep pin of the XB24 to read the current mode through a voltage level as the Section 3.3 illustrated.

For the XB24 firmware, we can choose between two different types depending on the data packaging:

- AT firmware. Using this firmware, the device is configured to send the information by AT commands. All the data read in the XB24 UART port will be sent depending on some initial configurations that must be defined by the user. The communication is done in a serial fashion.
- API firmware. Using this firmware, the information is sent and received in a package form. Therefore, the XB24 will send different packages depending on the content reading in the UART, which is supported by a specific protocol defined by the manufacturer.

Considering the differences between both firmwares, on the one hand, the AT firmware is chosen for the XB24 end-device (sensor motes) because the transmission size is smaller than the transmission on the API firmware. On the other and, the API firmware is chosen for the XB24 coordinator (collector mote) because the package form enables to identify the sender.

**Figure 4:**
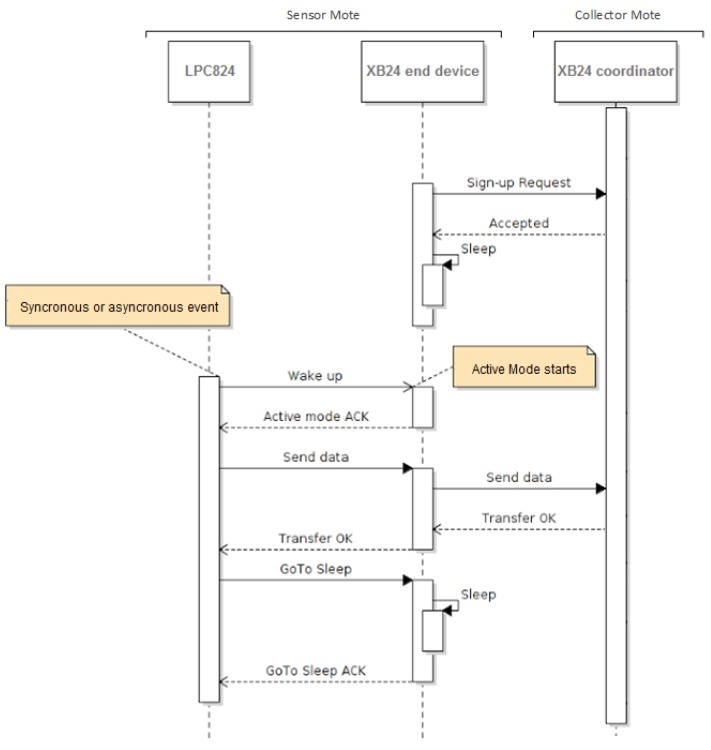
Sequence diagram of LOw power Konsumption Algorithm (LOKA) to reduce consumption in a sensor mote. It is divided into three columns that correspond to the execution flow of the LPC824 program, the XB24 end-device program and the XB24 coordinator program.

In this order, the Figure 4 illustrates the time-to-time that each element of a sensor mote is in low power consumption mode or in active mode as well as the exchange of data between the sensor mote and the collector mote. Thereupon, the Diagram of Figure 4 is explained *column by column* analyzing the program flow of each device taking into account that the LPC824 and the XB24 end-device correspond to the sensor mote and the XB24 coordinator corresponds to the collector mote.

In respect of the first column, initially, the LPC824 is in *Power down* mode. It will wake up and change to active mode when the wake-up pin value changes due to an alarm detection or time out. Both events raise an exception in the microcontroller that should be handled with the highest priority. As consequence, it reads the values of the sensor’s variables. After that, the LPC824 sends a wake-up signal to the XB24 end-device Sleep_RQ pin and waits to detect the wake up signal reading by the On/Sleep pin of the XB24 end-device. When the XB24 is waked up, the LPC824 sends the information read by the UART to the XB24 end-device in order to be transmitted to the XB24 coordinator. When the wireless transmission is completed, the LPC824 commands the XB24 end-device to enter into *Sleep* mode using the XB24 Sleep_RQ pin. Finally, the LPC824 acknowledges the new mode of the XB24 end-device by means of the On/Sleep pin and sets itself to *Power-down* mode until a new exception is  detected.

The second columns corresponds to the XB24 end-device. Initially, the XB24 end-device is in *Sleep* mode after the initialization and it is joined to the WSN. The XB24 end-device will be in *Sleep* mode until it detects the wake up signal from the LPC824 in its Sleep_RQ pin. When it detects the signal, it changes to active mode reporting in the On/Sleep pin. At this moment, the XB24 end-device reads the information by the UART and sends it to the XB24 coordinator. Finally, it waits the sleep mode signal from the LPC824 and it will stay into sleep mode until a new transmission is needed.

The third column corresponds to the XB24 coordinator. It is always in active mode to maintain the network and to receive data from the sensor motes. The XB24 coordinator must support the *Sleep* mode of the XB24 end-devices without baning them from the network. This is achieved through the AT configuration using the Cyclic Sleep Period (SP) and Sleep options (SO). In our case SP corresponds to the minimum time of the cyclic WKT IRQ in the LPC824 of the sensor motes taking into account the low power clock WKT accuracy. Finally, SO configures some options for sleep.

LOKA’s pseudocode is shown in the Algorithm 1. The sensor mote code begins at line 17. At the start, the LPC824 commands the XB24 end-device to go to *Sleep* mode using the function *sleep_xb24* (lines 1–5). This function ensures that the XB24 is effectively in *sleep* mode. After that, the BOD device, the UART, the WKT, the wake up pin, the 10 kHz low power clock and their respective IRQs are initialized (line 19) for enabling to wake up the LPC824. Then, the LPC824 changes to *Power  down* mode (lines 21) and waits until an interruption triggers back it to the *active* mode. There are three possible IRQs: IRQ_WKT, IRQ_WAKE_UP_PIN and IRQ_BOD. When an IRQ is triggered, the LPC824 continues the execution. Each interruption has its own handler defined to clear its property flag (lines 11–16), if IRQ_BOD is triggered one flag is activated to send the battery level (lines 26,27). In that point, the mote reads the information of the sensors (line 23), commands the XB24 end-device to change to *active* mode using the function *wake_up_xb24* (lines 6–10) and sends the information to the collector mode through the XB24 end-device (line 25). Finally, the XB24 is set again to *Sleep* mode and the LPC824 changes itself to *Power down* mode repeating the  loop.

LOKA is a new approach in the newest DTNs since LOKA implements asynchronous package reception in the end-device for commanding operations while the end-device is in *Sleep* mode. To do this, LOKA can read these packets after the activation of the XB24 end-device (line 24). First, the message is saved in the output buffer of the sender. After that, the sender transmits to the XB24 end-device the message when the coordinator detects that the XB24 end-device is active (since LOKA has activated it changing its mode consumption). Then, LOKA incorporates the reading of the message (line 28) and, finally, the LPC824 carries out the required actions referred to the received packets (line 29).

**Algorithm 1** LOKA1: **function**
sleep_xb24()▹ Order to *sleep* XB242:  SLEEPRQ←TRUE▹ Signal to *sleep* XB243:  **while**
SLEEPSEL≠FALSE
**do**▹ Wait until XB24 is in *sleep* mode4:   nothing5:  **return**6: **function**
wake_up_xb24()▹ Order to wake up XB247:  SLEEPRQ←FALSE▹ Signal to wake up XB248:  **while**
SLEEPSEL≠TRUE
**do**▹ Wait until XB24 is in *active* mode9:   nothing10:  **return**11: **function**
handleIRQ_WKT▹ IRQ to send data at periodic interval12:  CLEAR_WKT_FLAG13: **function**
handleIRQ_WAKE_UP_PIN▹ IRQ to send data if an alarm14:  CLEAR_WAKE_UP_FLAG15: **function**
handleIRQ_BOD▹ IRQ if low battery level16:  IRQ_BOD←TRUE17: **procedure**
main▹ Sensor mote program18:  sleep_xb24()19:  INITIALIZEDEVICESANDIRQs▹ Initialize all used devices and IRQs20:  **loop**▹ Continuous operation21:   setPOWER_DOWN_MODE▹ Go to *low power* mode22:▹ Execution only continues after an IRQ triggers *active* mode23:   READ_ALL_SENSORS24:   wake_up_xb24()25:   UART_SEND▹ Send data samples to XB24 via UART26:   **if** IRQ_BOD **then**▹ Notify low battery level27:    UART_SEND_BATTERY_LEVEL28:   UART_READ▹ Read the received packets29:   Managementreceivedpackets▹ Carry out specific actions about the received packets30:   sleep_xb24()

## 5. Experiments

In order to analyze and compare the power consumption of a sensor mote that exploits our algorithm, we have built a sensor mote prototype in which we have implemented it and a second sensor prototype without it, i.e., it is always in active mode by default. We have not evaluated the package loss rate, the node connectivity errors or the transmission throughput since these parameters depend on the protocol, the radio and the environment, and our algorithm does not modify them. Then, we have assumed those parameters do no vary. Instead, our analysis focuses on the platform power consumption where LOKA is deployed.

The sensor mote prototype enables us to evaluate the impact of our algorithm in the power consumption of the mote. To provide a comprehensive point of view, this section is divided into three subsections: *Prototype Mote*, *Energy Measure* and *Comparative Between Platforms*. The first subsection details the sensor devices used for the sensor mote prototype. The second subsection explains how we have measured the energy consumption during operation and the result obtained for the energy consumption for the two prototype motes (with and without LOKA) in different operation scenarios. The third subsection compares our prototype with different platforms of the WSN state of the art that implement duty-cycling mechanisms.

For our experiments we have not compared our approach with other methods from the literature as we are exploiting a specific hardware that has not been used, to the best of our knowledge, in other similar works. In this regard, our algorithm is designed to work with the ZigBee protocol, so we cannot compare our approach with algorithms designed to work with other communication protocols. Moreover, some state of the art methods do not provide enough details to build prototypes to be evaluated.

### 5.1. Prototype Mote

This subsection presents the prototype sensor mote. In this sense, we have taken into account the requirements imposed by the electronic components described in the Section 3.3: the LPC824, the XB24, the battery and some sensors. This subsection goes into the electronic implementation details to allow that the prototype mote works as a whole. In this case, the prototype mote is divided into four parts:

- Circuit supply. It provides energy to power on the mote devices.
- Sensors devices. The sensors integrated in the mote: measure motion, temperature, humidity and light.
- The LPC824 connection. This item was discussed in Section 3.3. It implements the logic of the sensor mote.
- The XB24 socket. This item was also discussed in Section 3.3. It enables the communication using the ZigBee protocol.

**Figure 5:**
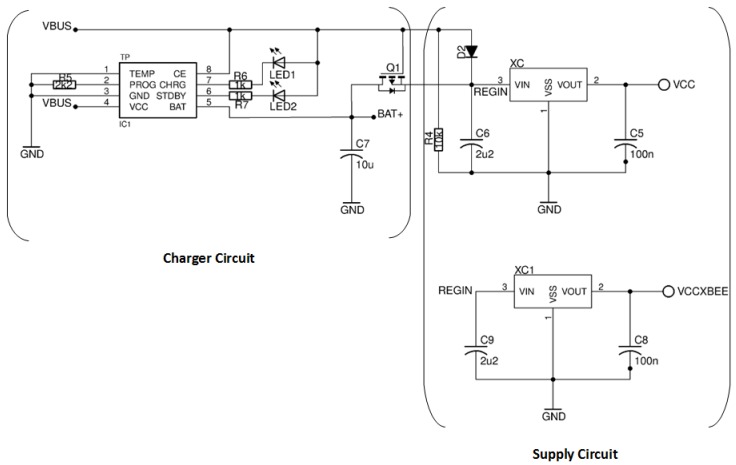
Charger and supply circuits.

For electricity grid independence, the motes are equipped with a 3.7 V lithium-ion (Li-Ion)battery. Related to the charger circuit, a standalone linear Li-ion single cell battery charger with thermal regulation (model TP4056) is included. It corresponds to the TP (or IC1) initials in the Figure 5. Also, the electronic components suggested by manufacturer are included.

Two leds are incorporated to display the charging state of the battery, R6 and R7. On the one hand, when the red led (R6) is on, the battery is charging. On the other hand, when the green led (R7) is on, the battery is full.

Regarding to the supply circuit, the prototype mote can be powered by a 5 V microUSB or by a 3.7 V Li-ion battery. Also, a MOSFET transistor is included for switching between the microUSB supply and the battery, allowing us to charge the Li-Ion battery in a safe way without interrupting the power supply. The MOSFET transistor included in the prototype mote meets this premise since the microUSB supply has higher priority than the Li-ion battery. That is, the MOSFET transistor is polarized when there is a microUSB supply. In this case, the Li-ion battery transistor is kept in isolation and the circuit powered by the microUSB is charging the battery at the same time. Otherwise, the circuit is powered by the Li-ion battery.

Also, there are two XC6206 voltage regulators after the connection at the supply power. One powers up the XB24 module since it has a high power demand while transmitting data. The second regulator powers up the other devices of the prototype mote.

On the sensor side, the prototype mote includes sensors to get samples with the values of some phenomenons. In our prototype we have integrated a HC-SR501 to detect motion, a DHT11 sensor to obtain the temperature and humidity and a Light Dependent Resistor (LDR) to measure luminosity.

The HC-SR501 is wired to the WAKE UP pin of the LPC824 with the objective of generating an event in LOKA when movement is detected. In reference to DHT11, it has is own digital communication protocol, so, it is wired to a digital IO of the LPC824 taking into account that the wire needs a pull-up resistor, R9, to ensure that the signal is inside a valid logic level. The LDR is included into the voltage divider. The LDR chosen is the PDV-P8103. The result of the voltage divider is passed to the ADC input of the microcontroller.

### 5.2. Energy Measure

The objective of this section is to evaluate the impact of LOKA in the energy consumption of a sensor mote. To do this, we have measured the energy consumption of our two prototype modules for a certain period of time in which both motes periodically send the sensors data to the collector mote (with and without LOKA). We have considered three different ways to measure the energy consumption contemplated in the state of art:

- By charging and discharging capacitors. Through the charge and discharge cycle of capacitors putting them between the supply and the prototype module as Jacob Andersen et al. [39] suggested. It was disapproved because it is an empirical solution that depends on the current leakages of the capacitors.
- By ammeter. Another possibility is to measure the current consumption periodically to know the energy consumption. However, an ammeter is not enough for our propose because the time between measures is too long and the burden voltage of common ammeters is very high, with the subsequent loss of resolution and accuracy.
- By shunt resistor and voltage amplifier. The last possibility is to measure the current consumption periodically jointing a shunt resistor and a low noise *chopper* amplifier. This is the solution that we have chosen because the time between measures and the precision of the measures are higher than with the previous solutions.

To implement the selected solution, we have used the commercial µCurrent device as Shuqin Geng et al. [40] suggest. We have combined it with the ADC of the LPC824 microcontroller to measure the energy consumption. Technically, the µCurrent device incorporates the shunt resistor and the *chopper* amplifier. On the one hand, the most important characteristics of µ Current are mA range of measure, 0.2% on µA measures accuracy and 0.5% on mA measure accuracy as well as 100 pA of resolution. On the other hand, the most important characteristics of the ADC of the LPC824 are 95KSPS (Kilo Samples Per Second) proved frequency sample and 0.366 mV LSB minimum.

The experiments with our prototype motes were carried out programing the WKT with different times (periodical transmit data interval), without movement (so wake up pin IRQ handle will never be activated), and with sufficient power battery so BOD IRQ handle will never be activated. In this way, the current of a sensor mote with LOKA and without LOKA is measured periodically. The Equation (1) calculates the energy assuming that the power supply (*E*) is constant and the time between the measures of the current (*T*) is periodic. If the time between measures is small enough, we can discretize the energy consumption as appear in the right term of the Equation (1).

(1) $$W\left[J\right]=\int_{1}^{2}Pdt=\int_{1}^{2}E\left(t\right)\;\cdot \;i\left(t\right)dt=E\;\cdot \;T\;\cdot \;\sum_{j=1}^{N}I_{j}$$

The Equation (2) corresponds to the ADC-LPC824 input where *n* is 12, the number of bits of the ADC and *result* is the output value of the ADC. We set the Vrefp and the Vrefn to 2.4 V and 0.9 V respectively. These values enable the maximum sensibility for the ADC-LPC824.

(2) $$I_{\mu Current}=V_{ADC}=\frac{result\;\cdot \;(V_{refp}-V_{refn})}{2^{n}}+V_{refn}$$

The Equation (3) corresponds to the total energy consumption in the prototype device taking into account the Equations (1) and (2).

(3) $$W\left[J\right]=\;\cdot \;E\;\cdot \;T\;\cdot \;\sum_{j=1}^{N}{\left[\frac{result_{j}\;\cdot \;\left(V_{refp}-V_{refn}\right)}{2^{n}}+V_{refn}\right]}_{j}=\frac{E}{f}\;\cdot \;\left[t\;\cdot \;f\;\cdot \;V_{refn}+\frac{V_{refp}-V_{refn}}{2^{n}}\;\cdot \;\sum_{j=1}^{N}result_{j}\right]$$

Finally, we set the following setting values for the Equation (3): *E* to 3.7 V, *Vrefp* to 2.4 V, *Vrefn* to 0.9 V and a frequency of 95 KSPS. Then, Equation (4) shows the consumption in Joules for a time *t* gathering *N* energy measures.

(4) $$W\left[J\right]=3895\;\times \;10^{-8}\;\cdot \;\left(85500\;\cdot \;t+\frac{1}{2730}\;\cdot \;\sum_{j=1}^{N}result_{j}\right)$$

**Figure 6:**
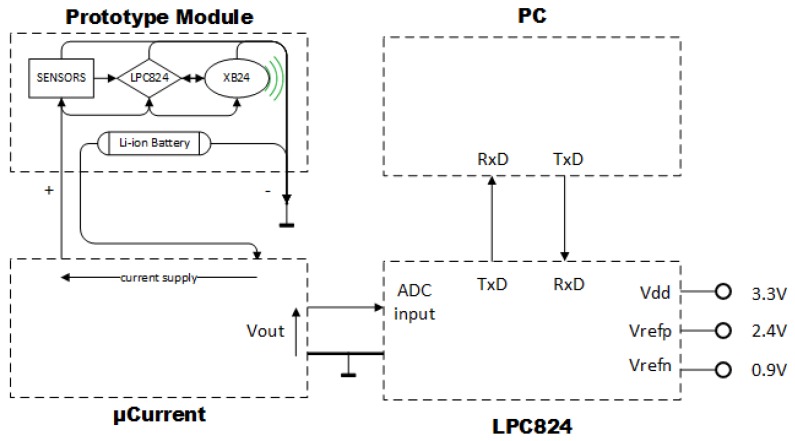
Experimental energy measuring schema is measured converting the current into voltage with the µ Current device and using it as the input of the LPC824’s ADC to be calculated with a PC.

The experimental setup is shown in Figure 6. It is composed of a µCurrent device, the LPC824 and a PC. Through this configuration, we implemented the Equation (4). LPC824 calculates the summation for *N* measures of the ADC at a frequency of 95KSPS. Then, it sends to a PC the summation through the UART port. Finally, the PC calculates the Equation (4) and saves the result.

We have collected measures with different configurations of transmit data interval and operation time. The collected measures are divided into two classes of experiments: without LOKA and with LOKA. In the experiments without LOKA there is no duty-cycling algorithm implemented in the sensor mote, and therefore, all electronics components are in active mode. In the experiments with LOKA, the designed duty-cycling algorithm turn off and on the radio module (XB24 end-device) and change the power mode of the electronic components.

These experiments always send periodically 64 bytes of sensor data to analyze the energy consumption during the operation time. One one side, the prototype mote with LOKA was tested in 360 experiments divided in blocks of 30 measures. On the other side, the prototype mote with LOKA was tested in 60 experiments divided in blocks of 5 measures. The energy consumption obtained showed that the standard deviation is small so we consider that the number of samples is enough to support our conclusions. For both experiments, we considered different values for the transmit interval time and the operation time:

- Discrete values of transmit data interval: [10, 30, 60] (seconds).
- Discrete values of operation time: [60, 300, 600, 900] (seconds).

**Figure 7:**
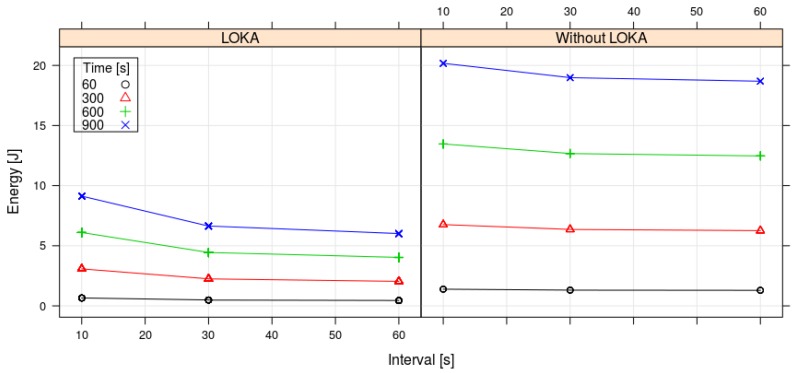
Results for the energy consumption, transmission time and interval with LOKA and without LOKA. Each point in the figure actually contains 30 or 5 measures; the low variability makes them appear in the figure like a single point.

**Figure 8:**
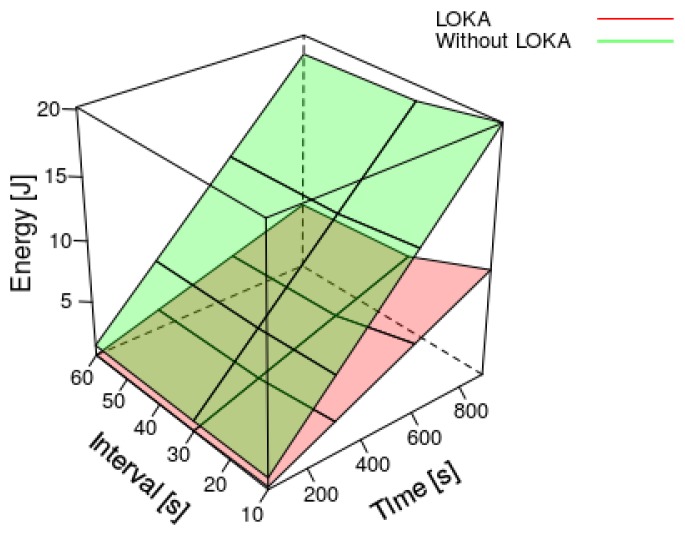
Results for the energy consumption of our experiments.

As can be observed in the Figure 7 and Figure 8, the energy consumption is reduced along the time using the LOKA. The longer the operation time and the longer the transmit data interval, the higher consumption reduction we obtain with LOKA. The maximum energy consumption reduction in our experiments was applying LOKA with a transmit data interval of 60 s and a time operation of 900 s getting a reduction above 12 Jules. In contrast, the minimum energy consumption reduction was applying LOKA with a transmit data interval of 10 s and a time operation of 60 s getting a reduction below 1 Joule. In all cases, applying the Equation (5), LOKA always reduces the energy consumption more than 55% respect to the prototype without our algorithm. Then, we achieve our objective of extending the time between battery charges.

(5) $$Energy\;reduction=\frac{E_{without\;LOKA}-E_{with\;LOKA}}{E_{without\;LOKA}}\times 100\%$$

### 5.3. Comparison between LOKA and Popular WSN Platforms

In this section the energy consumption of multiples academic and industrial WSN platforms are compared to the prototype where LOKA has been deployed. In particular, we focus on the energy consumption of the microcontrollers and the radio transceivers of these platforms.

Since replicating LOKA using other hardware platforms is expensive and time-consuming we have checked instead the energy consumption of these platforms in literature and compare them with the results we have gathered previously. Specially, we have chosen: *Mica2* [41], *Telos* [42], *TinyNode* [43] and *EyesIFX* [44]. The reason to use these platforms is they implement a duty-cycling mechanism documented in TinyOS [45] and their energy consumptions have been analyzed in the hardware data sheets.

While it is true that the energy consumption depends on many varying parameters e.g., MCUs frequency, CPUs architectures and duty-cycling mechanisms, the comparison establishes an actual reference where the energy consumption magnitudes are showed. Taking into account that all of them have similar hardware and a duty-cycling approach, this comparison displays LOKA’s prototype like a good alternative.

In the core of these platforms, *TinyNode*, *Telos* and *EyesIFX* are composed of a MSP430F1611, a 16-bits 8 MHz microcontroller and *Mica2* is composed of a ATmega128L, a 8-bits 8 MHz microcontroller. Our prototype uses a LPC824 microcontroller.

The radio transceivers are different: *Mica2* uses a TICC100 device, *Telos* a TICC2420, *TinyNode* a XE1205 and *EyexIFX* a TDA5250. Our prototype uses a XB24-Z7WIT-004.

As shown in the Table 1, the microcontroller where LOKA was deployed consumes less energy than the other microcontrollers. On the one hand, the energy consumption of the microcontroller in sleep mode is minimized 97% with respect to the MSP430F1611 microcontroller and 99% with respect to the ATmega128L microcontroller. On the other hand, the energy consumption of the microcontroller in active mode is minimized 39% with respect to the MSP430F1611 microcontroller and 86% with respect to the ATmega128L microcontroller.

In regard to the radio transceiver, the XB24 consumes more energy than the other radio transceivers since it has a flash memory inside and a powerful microcontroller to manage the ZigBee protocol. In the sleep mode case, the radio transceivers consume similar energy, yet the energy consumption can be tripled in the active mode case when the UART serial interface is used to send and receive information. It is not a problem since the time that LOKA uses the UART serial interface is very small. In regard to the active mode case without using UART serial interface, the XB24 manufacturer does not provide information.

Moreover, as can be noted from the Table 1, *Telos*, *TinyNode* and *EyesIFX* have a similar hardware energy consumption and *Mica2* has more consumption since its microcontroller energy consumption in active mode is very high (taking into account that all of them use the same software). This was pointed out by Henri et al. [43]. They checked the energy consumption at 1% duty-cycling mechanism, realizing that *Mica2* consumed 509 µW and *TinyNode* 489 µW.

These values were calculated without considering any sensor, therefore, the Equation (6) calculates an estimation of the real consumption along an operation time (t) using the sensors of the Section 5.1. The Table 2 shows the results for different operation times.

The Table 2 shows the energy consumption at 1% duty-cycling (D) mechanism, yet LOKA was tested at different D. Therefore, we need to analyze the results of the Section 5.2 to know the energy consumption at the same D. In this sense, the power in active mode can be inferred applying the Equation (8), where the energy consumption in sleep mode is deprecated. Then, the energy consumption at 1% D is obtained applying the Equation (9). Finally, the Table 2 shows the results for different operation times.

By comparing the energy consumption estimations of *Mica2* and *TinyNode* with our prototype, it can be seen that LOKA is more efficient that the TinyOs duty-cycling mechanism. The reason of less energy consumption applying LOKA than in the other platforms is because LOKA only uses interruption handles while TinyOs uses a computational abstraction layer. This layer wakes up the microcontroller CPU more frequently than applying only LOKA, and therefore, consumes more energy.

(6) $$W\left[J\right]=\left(P+13\times 10^{-3}\right)\;\cdot \;t$$

(7) $$W\left[J\right]=P_{active\;mode}\;\cdot \;t_{active\;mode}+P_{sleep\;mode}\;\cdot \;t_{sleep\;mode}=P_{active\;mode}\;\cdot \;\left(\frac{operation\;time}{transmit\;data\;interval}\right)$$

(8) $$P=W\left[J\right]\;\cdot \;\frac{transmit\;data\;interval}{operation\;time}$$

(9) $$W\left[J\right]=P_{active\;mode}\;\cdot \;\frac{D}{100}\;\cdot \;operation\;time$$

## 6. Conclusions

The Wireless Sensor Networks are useful in multiple scenarios where gathering data along a wide area is required. Often, this implies an electrical grid independence and wireless communications as the area to cover does not have the required infrastructure. Thus, WSNs are composed of several motes equipped with batteries and wireless transmission devices. The operation time of the WSN is dependent of the battery capacity and the logic implemented in the motes. In this regard, there are different studies about how to minimize the energy consumption: assessing the best transmission way, processing the data before transmit it or using wakeup-protocols with duty-cycling mechanism.

Following the last approach, this paper presents LOKA, a duty-cycling algorithm that reduces the energy consumption of each mote in the WSN by exploiting the power modes of the mote’s devices. To demonstrate the efficiency gain using LOKA, we have deployed a prototype sensor mote with different sensors (presence, temperature, humidity and light) controlled by a Cortex M0+ microcontroller and equipped with a XB24 ZigBee communication module and a battery. Managing the power modes of the microcontroller and the XB24 module, in our experiments LOKA can reduce over 55% the energy consumption of the sensor motes. In future works, we want to study the impact of the combination of LOKA and adaptive algorithms in the context of energy harvesting systems and to compare our deployed prototype sensor with other new FRAM microcontrollers like MSP430FR5969.

## Figures and Tables

**Table 1:** WSN platforms hardware characteristics (from manufactures data sheet) working at 3.3 V.

Platforms	CPU			Radio			
	Model	Current Drain		Model	Current Drain		
		Sleep	Active		Sleep	Active	Using TX/RX
Mica2	ATmega128L	15 µA	8 mA	TI CC100	<1 µA	0.030–0.105 mA	10–27mA
Telos	MSP430F1611	5.1 µA	1.8 mA	TI CC2420	20 µA	0.426 mA	11–20 mA
TinyNode	MSP430F1611	5.1 µA	1.8 mA	XE1205	<1 µA	0.85 –1.10 mA	14–75 mA
EyesIFX	MSP430F1611	5.1 µA	1.8 mA	TDA5250	<1 µA	1.8 mA	9–12 mA
LOKA’s prototype	LPC824	0.15 µA	1.1 mA	XB24-Z7WIT-004	<1 µA	N/A	40 mA

**Table 2:** Average consumption estimation in Mica2, TinyNode and LOKA’s prototype using 1% duty-cycling mechanism.

Operation Time [s]	Consumption [J]		
	Mica2	TinyNode	LOKA Prototype
60	0.810	0.809	0.614
300	4.053	4.047	3.073
600	8.105	8.094	6.146
900	12.158	12.140	9.220
